# Supplementary material for: Psychobiotic Effects on Anxiety Are Modulated by Lifestyle Behaviors: A Randomized Placebo-Controlled Trial on Healthy Adults
Source: Nutrients. 2023 Mar 31;15(7):1706. doi: 10.3390/nu15071706 (PMC10096963; doi:10.3390/nu15071706)
Supplement: Supplementary file 1 [file nutrients-15-01706-s001.zip › Supplementary material S1_REV.pdf]

# Supplementary material

## Materials and methods

### Randomization effectiveness

In order to confirm that randomization was achieved successfully we calculated the Euclidean distance between each participant, in relation to their scores on the 10 scales. After generating the distance matrix, we ran a PCA over that matrix. As can be seen in **Figure S1a** there is no apparent clustering according to each group. Using the Wilcoxon rank-sum test we observed no difference between the placebo and treatment group on the first principal component ($W$ = 2320, $p$ = 0.86) nor on the second ($W$ = 2588, $p$= 0.17). To corroborate this conclusion we ran the same analysis but using the Bray-Curtis distance, a more robust measure of ecological distance [42] , and using a Principal Coordinate analysis (**Figure S1b**). Using the Wilcoxon rank-sum test we see no difference between the placebo and treatment group on the first principal coordinate (W = 2548, $p$ = 0.24) nor on the second ($W$ = 2218, $p$ = 0.79). As no clustering was observed between both groups at the baseline time, we confirmed the randomization process was performed successfully (See **Figure S1**)

### Lifestyle behavioral patterns

There were seven domains of lifestyle considered in this study: diet, physical activity, sleep behavior, nature exposure, social contact, social media use, and substance abuse. To reduce the dimensionality of the variables, we group them into three patterns of behaviors: healthy and risky behaviors. We also grouped some domains in a third category named undetermined behaviors. In the healthy behaviors pattern, a higher score represents health benefits. In the risk behaviors pattern, on the contrary, a higher score indicates detrimental effects of the behaviors. The “undetermined category” points to those categories that don't fit into “higher is best” or “higher is worst” parameters according to the selected references.

Healthy behavior patterns include the diet domain (whole grains, fruits, vegetables, omega 3, fermented foods), physical activity, nature exposure, and social contact. The dietary components of this category correspond to international recommendations (Carlos et al., 2018; Cena & Calder, 2020; National Health and Medical Research Council (Australia) & Australia. Department of Health

and Ageing., 2013; Ruano et al., 2013; US Department of Agriculture and US Department of Health and Human Services, 2020) and are also coherent with the well-studied Mediterranean diet and Mediterranean-like evidence-based healthy diet patterns (Cena & Calder, 2020). Risk behavior patterns include diet categories (red meat, milk, snacks, refined flour, and sugar), social media use, and substance abuse (alcohol and smoking). The foods included in this category are restricted or limited by the previously referenced international scientific consensus. Similarly, social media have been proven as a risk factor for mental well-being (Meier & Reinecke, 2021; Odgers & Jensen, 2020), while alcohol and smoking behaviors are known as health-detrimental behaviors. Undetermined behaviors include diet categories (oils, dairies, and eggs) and sleep behavior. The reason for including oils is that the item could be interpreted as processed vegetable oils - known as pro-inflammatory- and also as olive oil - which has known anti-inflammatory effects. The dairy's item responses could include wild-fermented cheese and probiotic yogurt, which are part of the Mediterranean diet recommendations, and processed sugary yogurts, cheeses, and similar products that fit the category of processed foods and therefore health detrimental. A summary table of this categorization can be seen in **Table S1.**

To understand the relationship between lifestyle habits and psychological variables we reduced the dimensionality of the lifestyle variables using PCA. Considering that health-conscious behaviors are usually adopted in clusters [43], we grouped lifestyle variables based on available evidence, by whether they belonged to healthy, risky, or uncertain behaviors and ran a separate PCA for each of the three groups of variables. The first component of the variables comprising healthy behaviors explained 25.9% of the variability of that set of variables, the one for risk behaviors explained 24.6% of the variability of that set of variables, and the one for uncertain behaviors explained 42.5% of the variability of that set of variables. As expected, for health and risk behavior all the variables that comprised each group loaded in the same direction on the first component of the PCA, indicating that they were correlated, and confirming our prior formulation is backed up by our dataset **(Figure S2 for details)**.

After running the PCA of lifestyle habits, we generated principal component scores for each subject. While the first component of a PCA represents the dimension that better captures the variability of a set of variables, the principal component score of the first dimension is a linear combination of that set of variables which represents the projected score of the subject in the first component. The component scores of the set of variables comprising healthy behaviors will be called “Health Behavior” (HB), the one from the set of variables comprising risk behaviors will be called “Risk

Behavior" (RB) and the one from the set of variables comprising uncertain behaviors will be called "Uncertain behavior" (UB). Each of these three variables is a value that summarizes the lifestyle habits of each subject, according to the three categories of lifestyle habits.

Since lifestyle data was measured as several ordinal variables, we repeated the PCA procedure stated above, but with categorical PCA [38]. The correlation between the classical PCA scores and categorical PCA scores for the first principal components of Healthy (r(134) = 1, p < 0.001.), Risk (r(134) = 0.47, p < 0.001.), and uncertain (r(134) = -1, p < 0.001.) behaviors were all significant. Given their ease of interpretability and their correlation with categorical PCA scores, we will use classical PCA scores for the rest of the paper.

# Results

## Secondary outcomes

In SWLS there was a significant main effect of time F(2,246) = 7.76, p < 0.001), and a trend for the treatment (F(2,123) = 3.83, p = 0.053)), but not for their interaction **(Table 2)**. Post-hoc pairwise analysis showed significant increased scores at post-treatment (M diff = -0.82, t(123) = -3.36, p adjusted = 0.003) and follow-up (M diff = -0.91, t(123) = -3.04, p adjusted = 0.009) times compared to baseline. Between groups trend were not significant after post-hoc corrections. No effects on time, treatment or time*treatment was observed for Positive Affect (PANAS_POS**, Table 2).** For Negative Affect (PANAS_NEG, **Table 2**), we observed a significant main effect of time (F(2,248) = 3.17, p = 0.044) and time*treatment (F(2,248) = 3.53, p = 0.031), as well as a trend for treatment (F(2,124) = 3.55, p = 0.062). After post-hoc analysis, an increased score in the follow-up time point compared to post-treatment time point was observed (M diff = -1.54, t(124) = -2.82, p adjusted = 0.017). Additionally, we observed a significant difference between groups at baseline that were no significant after a Bonferroni Correction (M diff = 4.12, t(124) = 2.62, p = 0.010, p adjusted = 0.146). However, this trend makes us suspect that the implementation of the instrument in this experiment may not have been entirely successful, so we will discuss it with reservations. In the STAI scale we observed only a significant main effect of time (F(2,250) = 19.11, p < 0.001. **Table 2**). This effect was maintained after post-hoc correction between baseline and post-treatment times (M diff = -4.022,

t(125) = -5.560, p adjusted < 0.001) and between baseline and follow-up times (M diff = -3.467, t(125) = -5.111, p adjusted <0.001). For SF-36 (mental and physical) and DERS, the results followed similar patterns in which only a main effect of time was observed (SF36_MEN, F(2,248) = 6.827, p = 0.001; SF36_PHY, F(2,244) = 5.29, p < 0.006; DERS, F(2,244) = 12.9, p < 0.001, **Table 2**). Post-hoc analysis revealed a significant increased score in the mental component of the SF-36 at post-treatment (M diff = -3.881, t(124) = -3.46, p adjusted = 0.002) and follow-up (M diff = -3.396, t(124) = -2.644, p adjusted = 0.028) times compared to baseline. For the physical component of the SF-36, the only significant difference observed after post-hoc correction was between baseline and post-treatment times (M diff = -2.25, t(118) = -3.30, p adjusted =0.004) Finally, Bonferroni corrected post-hoc analysis revealed that DERS scores at post-treatment (M diff = 2.7, t(122) = 3.65, p adjusted =0.001) and follow-up (M diff = 4.04, t(122) = 4.04, p adjusted < 0.001) times were significantly lower than baseline.

## Exploratory outcomes

MAIA overall scores indicated a main effect of time(F(2,250) = 7.27, p < 0.001. **Table 2**). Post-hoc analysis revealed increased scores in both post-treatment (M diff = -0.14, t(125) = -2.88, p adjusted =0.014) and follow-up times (M diff = -0.15, t(125) = -3.06, p adjusted =0.008) compared baseline. The same effects were observed for FFMQ scale (Time effects, F(2,248) = 6.84, p = 0.001. **Table 2**), reflected in the increased FFMQ scores after post-treatment (M diff = -3.05, t(124) = -2.52, p adjusted =0.039) and follow-up times (M diff = -4.22, t(124) = -3.06, p adjusted =0.008) compared baseline.

# Tables

## Table S1. Lifestyle behaviors, items, and responses.

| | Question | Responses N° (% of respondents) | | | |
|---|---|---|---|---|---|
| | | 0 | 1-3 | 4-6 | 6 or more |
| 1 | How many times a day do you smoke? | 121 (89) | 12 (9) | 2 (1) | 1 (1) |
| | | 0 | 1 | 2 - 3 | 4 or more |
| 2 | How many portions of fresh fruits (such as: apple, pear, banana, melon, others) do you consume per day? | 9 (7) | 56 (41) | 64 (47) | 7 (5) |
| 3 | How many portions of fresh vegetables -or cooked- (such as: broccoli, spinach, tomates, asparagus, others) do you consume per day? | 2 (1) | 33 (24) | 73 (54) | 28 (21) |
| 4 | How many portions of whole grains (such as: potato -not fried-, beans, chickpea, others) do you consume per day? | 6 (4) | 71 (52) | 51 (38) | 8 (6) |
| 5 | How many portions of fermented products (such as: chucrut, kombucha, kimchi, others) do you consume per day? | 73 (54) | 48 (35) | 13 (10) | 2 (1) |
| 6 | How many times a month do you visit some of these places: natural parks, hills, woods? | 50 (37) | 46 (34) | 12 (9) | 28 (21) |
| | | 0 | 1 - 2 | 3 - 5 | 6 or more |
| 7 | How many portions of dairy products (such as: cheese, butter, ice-cream, yogurt, others) do you consume per week? | 48 (35) | 47 (35) | 29 (21) | 12 (9) |
| 8 | How many days a week do you eat eggs or use it as ingredients for cooking? | 45 (33) | 52 (38) | 28 (21) | 11 (8) |
| 9 | How many days a week do you drink animal milk/cream or add it to your meals? | 96 (71) | 26 (19) | 9 (7) | 5 (4) |
| 10 | How many of your weekly meals include animal meat (such as: beef, chicken, fish, pork, lamb, turkey, others)? | 97 (71) | 16 (12) | 16 (12) | 7 (5) |
| 11 | How many of your semanal meals include vegetable oil, coconut oil, olive oil, canola oil (others)? | 12 (9) | 14 (10) | 40 (29) | 70 (51) |
| | | 0 | 1 - 2 | 3 - 5 | 6 - 7 |

| | | | | | |
|---|---|---|---|---|---|
| 12 | How many times per week do you consume alcohol, wine, beer, cocktails (others)? | 44 (32) | 76 (56) | 16 (12) | 0 (0) |
| | | **Minimum** | **Little** | **Medium** | **High** |
| 13 | How do you describe your consumption level of white flour, bread, pasta, cakes, cookies (others) | 49 (36) | 31 (23) | 40 (29) | 16 (12) |
| 14 | How do you describe your consumption level of unhealthy products (such as: processed snacks/sweet or salty-)? | 65 (48) | 40 (29) | 24 (18) | 7 (5) |
| | | **No** | **Improbable** | **Posible** | **Yes** |
| 15 | Do you think that you are obtaining all the Omega-3 you need from sources like lino seeds, nuts, sesame seeds and chia? | 9 (7) | 26 (19) | 69 (51) | 32 (24) |
| | | **Much** | **Quite** | **Not Much** | **Nothing** |
| 16 | Are you serious about eliminating added sugar in your home and the products that you buy? | 5 (4) | 29 (21) | 56 (41) | 46 (34) |
| | | **0** | **1 - 2** | **3 - 5** | **6 or more** |
| 17 | How many times per week do you practice any physical activity for more than 30 minutes? | 27 (20) | 33 (24) | 65 (48) | 11 (8) |
| | | **0** | **1 - 2** | **3 - 5** | **6 - 7** |
| 18 | How many times per week do you spend, at least, between 30 and 60 minutes working in your interpersonal relationships (friends, family, others)? | 4 (3) | 56 (41) | 53 (39) | 23 (17) |
| | | **less than 6** | **6 - 7** | **7 - 9** | **9 or more** |
| 19 | How much time do you sleep, uninterruptedly, each day? | 18 (13) | 74 (54) | 43 (32) | 1 (1) |
| | | **0** | **less than 1** | **1 - 2** | **2 or more** |
| 20 | How many hours per day do you spend in social media apps (such as: Instagram, Facebook, WhatsApp, Twitter, others)? | 1 (1) | 8 (6) | 42 (57) | 51 (70) |

**Table S2.** Baseline measurements.

| Group | Placebo<br>n = 67<br>Mean (std) | Probiotic<br>n = 68<br>Mean (std) | *p* |
|---|---|---|---|
| **Primary** | | | |
| RYFF | 4.38 (0.5) | 4.46 (0.54) | *0.38* |
| **Secondary** | | | |
| SWLS | 19.54 (3.84) | 20.15 (4.16) | *0.26 (mw)* |
| PANAS_Pos | 38.07 (6.15) | 38.99 (37.33) | *0.42* |
| PANAS_Neg | 22.93 (10.02) | 19.78 (8.44) | *0.09 (mw)* |
| STAI | 11.06 (5.78) | 9.34 (5.83) | *0.09* |
| SF36-P | 84.22 (11.36) | 84.24 (87.12) | *0.82 (mw)* |
| SF36-M | 70.26 (15.97) | 71.85 (16.11) | *0.40 (mw)* |
| DERS | 47.40 (12.44) | 46.78 (13.59) | *0.54 (mw)* |
| **Exploratory** | | | |
| MAIA | 3.12 (0.62) | 3.28 (0.8) | *0.4* |
| FFMQ | 136.4 (17.94) | 137.8 (21.2) | *0.68* |

Abbreviations: RYFF, Ryff Well-being Scale; STAI, State Trait Anxiety Inventory; SWLS, Satisfaction with Life Scale;; PANAS_Pos, Positive and Negative Affect Scale (Positive); PANAS_Neg, Positive and Negative Affect Scale (Negative); SF36-P, Short Form Health Survey (Physical); SF36-M, Short Form Health Survey (Mental); DERS, Difficulties in Emotional Regulation Scale; MAIA, Multidimensional Assessment of Interoceptive Awareness; FFMQ, Five Facet Mindfulness Questionnaire
*p value is the results of Student's t-test or Mann-Whitney U test (mw).*

**Table S3.** Correlation between lifestyle variables and psychological variables.

| Variable | *M* | *SD* | 1 | 2 | 3 | 4 | 5 | 6 | 7 | 8 | 9 | 10 | 11 | 12 |
|---|---|---|---|---|---|---|---|---|---|---|---|---|---|---|
| 1. Health Behaviors | 0.01 | 1.44 | | | | | | | | | | | | |
| 2. Risk Behaviors | 0.01 | 1.41 | -0.42** [-0.55, -0.27] | | | | | | | | | | | |
| 3. Undetermined behaviors | 0.01 | 1.31 | -0.17 [-0.33, 0.00] | 0.41** [0.26, 0.54] | | | | | | | | | | |
| 4. DERS | 46.41 | 11.81 | -0.22** [-0.38, -0.06] | 0.14 [-0.03, 0.31] | -0.01 [-0.18, 0.17] | | | | | | | | | |
| 5. RYFF | 4.42 | 0.52 | 0.34** [0.18, 0.48] | -0.27** [-0.42, -0.10] | -0.11 [-0.27, 0.06] | -0.63** [-0.72, -0.51] | | | | | | | | |
| 6. SWLS | 19.95 | 3.83 | 0.28** [0.12, 0.43] | -0.13 [-0.29, 0.04] | 0.05 [-0.12, 0.22] | -0.50** [-0.62, -0.36] | 0.72** [0.63, 0.80] | | | | | | | |
| 7. STAI | 10.19 | 5.85 | -0.43** [-0.56, -0.28] | 0.30** [0.13, 0.44] | 0.03 [-0.14, 0.20] | 0.45** [0.30, 0.58] | -0.70** [-0.78, -0.61] | -0.59** [-0.69, -0.46] | | | | | | |
| 8. PANAS_POS | 38.53 | 6.49 | 0.41** [0.25, 0.54] | -0.14 [-0.30, 0.03] | 0.02 [-0.15, 0.19] | -0.35** [-0.49, -0.19] | 0.57** [0.44, 0.67] | 0.45** [0.30, 0.57] | -0.62** [-0.72, -0.51] | | | | | |
| 9. PANAS_NEG | 21.34 | 9.36 | -0.27** [-0.42, -0.10] | 0.25** [0.08, 0.40] | 0.08 [-0.09, 0.25] | 0.46** [0.31, 0.58] | -0.54** [-0.65, -0.40] | -0.40** [-0.53, -0.25] | 0.61** [0.49, 0.71] | -0.13 [-0.29, 0.04] | | | | |
| 10. MAIA | 3.22 | 0.72 | 0.40** [0.25, 0.54] | -0.30** [-0.44, -0.13] | -0.10 [-0.26, 0.07] | -0.41** [-0.54, -0.26] | 0.46** [0.31, 0.58] | 0.23** [0.07, 0.39] | -0.43** [-0.56, -0.29] | 0.38** [0.23, 0.52] | -0.40** [-0.53, -0.24] | | | |
| 11. FFMQ | 137.13 | 19.58 | 0.39** [0.24, 0.53] | -0.34** [-0.48, -0.18] | -0.15 [-0.31, 0.02] | -0.66** [-0.74, -0.55] | 0.60** [0.48, 0.70] | 0.37** [0.21, 0.50] | -0.55** [-0.66, -0.42] | 0.43** [0.28, 0.56] | -0.54** [-0.65, -0.40] | 0.70** [0.60, 0.78] | | |
| 12. SF36_PHY | 85.13 | 10.03 | 0.44** [0.29, 0.56] | -0.19* [-0.35, -0.02] | -0.07 [-0.24, 0.10] | -0.27** [-0.42, -0.10] | 0.31** [0.14, 0.45] | 0.22* [0.05, 0.38] | -0.41** [-0.54, -0.25] | 0.37** [0.22, 0.51] | -0.26** [-0.42, -0.10] | 0.28** [0.11, 0.43] | 0.31** [0.15, 0.46] | |
| 13. SF36_MEN | 71.06 | 16.00 | 0.46** [0.32, 0.58] | -0.29** [-0.44, -0.13] | -0.04 [-0.21, 0.13] | -0.47** [-0.59, -0.32] | 0.52** [0.38, 0.63] | 0.47** [0.33, 0.59] | -0.63** [-0.72, -0.52] | 0.49** [0.35, 0.61] | -0.48** [-0.60, -0.34] | 0.37** [0.21, 0.51] | 0.48** [0.34, 0.60] | 0.62** [0.50, 0.71] |

*Note. M* and *SD* are used to represent mean and standard deviation, respectively. Values in square brackets indicate the 95% confidence interval for each correlation. The confidence interval is a plausible range of population correlations that could have caused the sample correlation (Cumming, 2014). * indicates $p < 0.05$. ** indicates $p < 0.01$.

**Table S4.** Health behavior over time.

| | Health | | | | | |
|---|---|---|---|---|---|---|
| *Predictors* | *beta coeff.* | *Std. Error* | *CI* | *t statistic* | *df* | *p* |
| (Intercept) | -0.08 | 0.19 | -0.46 – 0.30 | -0.42 | 187.27 | 0.677 |
| Sexo [M] | 0.27 | 0.29 | -0.30 – 0.85 | 0.94 | 131.13 | 0.349 |
| Edad | 0.01 | 0.02 | -0.02 – 0.04 | 0.52 | 130.57 | 0.602 |
| Probiotico [Treatment] | 0.05 | 0.26 | -0.46 – 0.56 | 0.20 | 193.71 | 0.844 |
| Time [Post] | 0.04 | 0.14 | -0.24 – 0.32 | 0.28 | 254.96 | 0.780 |
| Time [fw] | 0.01 | 0.15 | -0.27 – 0.30 | 0.10 | 256.48 | 0.919 |
| Probiotico [Treatment] × Time [Post] | -0.06 | 0.20 | -0.45 – 0.33 | -0.32 | 254.67 | 0.752 |
| Probiotico [Treatment] × Time [fw] | -0.03 | 0.20 | -0.43 – 0.37 | -0.13 | 255.89 | 0.899 |
| **Random Effects** | | | | | | |
| $\sigma^2$ | 0.66 | | | | | |
| $\tau_{00\ Subject}$ | 1.61 | | | | | |
| ICC | 0.71 | | | | | |
| $N_{Subject}$ | 135 | | | | | |
| Observations | 393 | | | | | |
| Marginal $R^2$ / Conditional $R^2$ | 0.007 / 0.712 | | | | | |

**Table S5.** Risky behavior over time.

| | Risky Behavior | | | | | |
|---|---|---|---|---|---|---|
| *Predictors* | *beta coeff.* | *Std. Error* | *CI* | *t statistic* | *df* | *p* |
| (Intercept) | -0.10 | 0.18 | -0.45 – 0.25 | -0.57 | 181.77 | 0.570 |
| Sexo [M] | 0.19 | 0.27 | -0.36 – 0.73 | 0.68 | 131.12 | 0.496 |
| Edad | -0.02 | 0.01 | -0.05 – 0.01 | -1.50 | 130.61 | 0.137 |
| Probiotico [Treatment] | 0.13 | 0.24 | -0.35 – 0.60 | 0.52 | 187.60 | 0.603 |
| Time [Post] | 0.07 | 0.13 | -0.18 – 0.31 | 0.53 | 254.89 | 0.597 |
| Time [fw] | 0.14 | 0.13 | -0.11 – 0.40 | 1.12 | 256.27 | 0.265 |
| Probiotico [Treatment] × Time [Post] | -0.14 | 0.18 | -0.49 – 0.21 | -0.80 | 254.61 | 0.427 |
| Probiotico [Treatment] × Time [fw] | -0.30 | 0.18 | -0.65 – 0.06 | -1.64 | 255.73 | 0.102 |
| **Random Effects** | | | | | | |
| $\sigma^2$ | 0.52 | | | | | |
| $\tau_{00\ Subject}$ | 1.43 | | | | | |
| ICC | 0.73 | | | | | |
| $N_{Subject}$ | 135 | | | | | |
| Observations | 393 | | | | | |
| Marginal $R^2$ / Conditional $R^2$ | 0.017 / 0.737 | | | | | |

**Figure S1. Randomization effectiveness.** Randomization success was evaluated by calculating the PCA over Euclidean distances (a) or Principal coordinate analysis over the Bray-Curtis distance (b) between subjects.

**Figure S2.** Depiction of the first to principal components of (a) Health behaviors, (b) Risk behaviors, and (c) uncertain behaviors. In A and B, all the variables charge with a positive value on the first dimension, indicating a correlation between them.

**Figure S3.** Linear Regression predicted scores using the linear regression model " Scales ~ Sex + Age + HB + Treatment+ HB*treatment + HB*treatment*time". Red and blue colors represent placebo and probiotic groups, respectively. Points represent the values for RYFF (a), SWLS (b), PANAS_POS (c), PANAS_NEG (d), SF36_MEN (e), SF36_PHY(f), MAIA (g). The shaded region represents the standard error.
