# Supplementary material for: Psychobiotic Effects on Anxiety Are Modulated by Lifestyle Behaviors: A Randomized Placebo-Controlled Trial on Healthy Adults
Source: Nutrients. 2023 Mar 31;15(7):1706. doi: 10.3390/nu15071706 (PMC10096963; doi:10.3390/nu15071706)
Supplement: Supplementary file 1 [file nutrients-15-01706-s001.zip › Supplementary material S2_REV.pdf]

Table S6. Three-way interaction model for healthy behavior in RYFF.

	RYFF					
Predictors	*beta coeff.*	*Std. Error*	*CI*	*t statistic*	*df*	*p*
(Intercept)	-0.03	0.07	-0.17 – 0.10	-0.51	185.52	0.611
Sex [Male]	0.02	0.10	-0.18 – 0.23	0.21	129.25	0.836
Age	0.02	0.01	0.01 – 0.03	3.32	129.01	0.001
Probiotic [Treatment]	0.08	0.09	-0.10 – 0.26	0.91	192.03	0.366
Health	0.09	0.04	0.01 – 0.18	2.20	191.58	0.029
Time [Post]	-0.02	0.05	-0.12 – 0.07	-0.45	255.02	0.650
Time [fw]	-0.06	0.05	-0.16 – 0.04	-1.16	256.29	0.246
Probiotic [Treatment] × Health	0.05	0.06	-0.08 – 0.18	0.75	192.06	0.452
Probiotic [Treatment] × Time [Post]	0.02	0.07	-0.12 – 0.16	0.26	255.02	0.795
Probiotic [Treatment] × Time [fw]	0.09	0.07	-0.05 – 0.23	1.33	256.12	0.185
Health × Time [Post]	-0.04	0.03	-0.10 – 0.03	-1.11	255.02	0.270
Health ×Time [fw]	-0.01	0.03	-0.07 – 0.06	-0.16	255.49	0.873
(Probiotic [Treatment] × Health) ×Time [Post]	0.06	0.05	-0.04 – 0.15	1.17	255.02	0.245
(Probiotic [Treatment] × Health) × Time [fw]	0.06	0.05	-0.04 – 0.16	1.24	255.24	0.216

Random Effects

σ^2	0.08
$\tau_{00\ Subject}$	0.20
ICC	0.71
$N_{Subject}$	135
Observations	398
Marginal R^2 / Conditional R^2	0.174 / 0.759

Table S7. Three-way interaction model for healthy behavior in SWLS.

	SWLS					
Predictors	*beta coeff.*	*Std. Error*	*CI*	*t statistic*	*df*	*p*
(Intercept)	-0.14	0.43	-0.99 – 0.70	-0.33	200.04	0.740
Sex [Male]	-0.52	0.64	-1.78 – 0.74	-0.81	128.94	0.417
Age	0.09	0.03	0.02 – 0.15	2.67	128.19	0.009
Probiotic [Treatment]	0.89	0.58	-0.25 – 2.04	1.53	210.69	0.126
Health	0.62	0.27	0.09 – 1.15	2.31	207.55	0.022
Time [Post]	-0.22	0.35	-0.91 – 0.46	-0.65	249.15	0.517
Time [fw]	-0.10	0.35	-0.80 – 0.60	-0.28	250.70	0.777
Probiotic [Treatment] × Health	0.09	0.41	-0.73 – 0.90	0.21	214.88	0.834
Probiotic [Treatment] × Time [Post]	0.39	0.49	-0.58 – 1.36	0.79	249.38	0.430
Probiotic [Treatment] × Time [fw]	0.07	0.50	-0.92 – 1.06	0.13	252.13	0.895

Health × Time [Post]	-0.38	0.22	-0.82 – 0.06	-1.69	249.15	0.092
Health× Time [fw]	-0.37	0.23	-0.82 – 0.07	-1.64	249.72	0.102
(Probiotic [Treatment] × Health) × Time [Post]	0.52	0.35	-0.17 – 1.22	1.48	249.64	0.139
(Probiotic [Treatment] × Health) × Time [fw]	0.67	0.35	-0.03 – 1.36	1.88	253.06	0.061
**Random Effects**						
σ^2	4.02					
$\tau_{00\ Subject}$	7.28					
ICC	0.64					
$N_{Subject}$	135					
Observations	392					
Marginal R^2 / Conditional R^2	0.129 / 0.690					

Table S8. Three-way interaction model for healthy behavior in STAI.

	STAI					
Predictors	*beta coeff.*	*Std. Error*	*CI*	*t statistic*	*df*	*p*
(Intercept)	1.04	0.92	-0.78 – 2.86	1.12	273.38	0.263
Sex [Male]	-1.19	1.23	-3.63 – 1.25	-0.96	129.14	0.337
Age	-0.15	0.06	-0.28 – -0.02	-2.30	128.45	0.023
Probiotic [Treatment]	-1.71	1.26	-4.19 – 0.76	-1.36	286.81	0.174
Health	-1.65	0.58	-2.79 – -0.51	-2.84	285.92	0.005

Time [Post]	-0.66	0.98	-2.59 – 1.26	-0.68	253.94	0.498
Time [fw]	0.31	0.99	-1.64 – 2.27	0.31	256.04	0.754
Probiotic [Treatment] × Health	-0.02	0.89	-1.76 – 1.72	-0.02	286.87	0.981
Probiotic [Treatment] × Time [Post]	1.52	1.38	-1.20 – 4.23	1.10	253.99	0.272
Probiotic [Treatment] × Time [fw]	-0.31	1.40	-3.05 – 2.44	-0.22	255.65	0.827
Health × Time [Post]	0.59	0.65	-0.68 – 1.86	0.92	256.38	0.359
Health × Time [fw]	1.08	0.64	-0.17 – 2.33	1.70	254.22	0.090
(Probiotic [Treatment] × Health) × Time [Post]	-2.68	0.99	-4.64 – -0.73	-2.70	257.23	0.007
(Probiotic [Treatment] × Health) × Time [fw]	-1.90	0.97	-3.80 – 0.01	-1.96	253.66	0.051
**Random Effects**						
σ^2	31.71					
$\tau_{00\ Subject}$	21.67					
ICC	0.41					
$N_{Subject}$	135					
Observations	396					
Marginal R^2 / Conditional R^2	0.169 / 0.506					

Table S9. Three-way interaction model for healthy behavior in DERS.

	DERS					
Predictors	*beta coeff.*	*Std. Error*	*CI*	*t statistic*	*df*	*p*
(Intercept)	0.16	1.27	-2.34 – 2.67	0.13	217.80	0.898
Sex [Male]	-1.55	1.84	-5.18 – 2.08	-0.84	128.55	0.400
Age	-0.36	0.10	-0.55 – -0.17	-3.70	128.70	<0.001
Probiotic [Treatment]	-0.86	1.72	-4.24 – 2.53	-0.50	227.54	0.619
Health	-1.61	0.79	-3.17 – -0.05	-2.04	224.61	0.043
Time [Post]	0.42	1.11	-1.77 – 2.61	0.38	249.99	0.705
Time [fw]	0.05	1.14	-2.19 – 2.29	0.05	251.87	0.963
Probiotic [Treatment] × Health	-0.27	1.21	-2.65 – 2.11	-0.23	226.36	0.821
Probiotic [Treatment] × Time [Post]	0.22	1.57	-2.88 – 3.32	0.14	250.31	0.888
Probiotic [Treatment] × Time [fw]	0.57	1.59	-2.57 – 3.71	0.36	251.42	0.721
Health × Time [Post]	0.54	0.71	-0.87 – 1.94	0.75	249.18	0.454
Health × Time [fw]	0.36	0.72	-1.06 – 1.79	0.50	250.09	0.615
(Probiotic [Treatment] × Health) × Time [Post]	-2.75	1.11	-4.95 – -0.56	-2.47	250.87	0.014
(Probiotic [Treatment] × Health) × Time [fw]	-1.98	1.10	-4.14 – 0.19	-1.80	249.77	0.074

Random Effects

σ^2	40.56
$\tau_{00\ Subject}$	58.12
ICC	0.59
$N_{Subject}$	135
Observations	392
Marginal R^2 / Conditional R^2	0.176 / 0.661

Table S10. Three-way interaction model for healthy behavior in PANAS_POS.

	PANAS_POS					
Predictors	*beta coeff.*	*Std. Error*	*CI*	*t statistic*	*df*	*p*
(Intercept)	-0.56	0.80	-2.13 – 1.01	-0.70	293.46	0.484
Sex [Male]	1.21	1.03	-0.83 – 3.25	1.17	129.20	0.242
Age	0.07	0.05	-0.03 – 0.18	1.39	128.69	0.167
Probiotic [Treatment]	0.82	1.09	-1.32 – 2.96	0.75	307.31	0.454
Health	0.93	0.50	-0.05 – 1.92	1.86	306.40	0.064
Time [Post]	-0.51	0.89	-2.25 – 1.24	-0.57	255.80	0.568
Time [fw]	-0.30	0.90	-2.07 – 1.47	-0.33	258.20	0.743
Probiotic [Treatment] ×× Health	1.97	0.77	0.47 – 3.48	2.58	307.37	0.010
Probiotic [Treatment] ×× Time [Post]	1.06	1.24	-1.39 – 3.51	0.85	255.07	0.395

Probiotic [Treatment] ×× Time [fw]	0.17	1.26	-2.32 – 2.66	0.13	257.45	0.895
Health × Time [Post]	0.71	0.57	-0.43 – 1.84	1.23	256.10	0.220
Health × Time [fw]	0.06	0.58	-1.08 – 1.19	0.10	256.31	0.921
(Probiotic [Treatment] × Health) × Time [Post]	-0.32	0.88	-2.05 – 1.40	-0.37	255.09	0.714
(Probiotic [Treatment] × Health) × Time [fw]	0.01	0.88	-1.72 – 1.74	0.01	255.23	0.989
**Random Effects**						
σ^2	25.99					
$\tau_{00\ Subject}$	13.86					
ICC	0.35					
$N_{Subject}$	135					
Observations	397					
Marginal R^2 / Conditional R^2	0.227 / 0.496					

Table S11. Three-way interaction model for healthy behavior in PANAS_NEG.

	**PANAS_NEG**					
Predictors	*beta coeff.*	*Std. Error*	*CI*	*t statistic*	*df*	*p*
(Intercept)	1.23	0.95	-0.65 – 3.12	1.29	206.55	0.197
Sex [Male]	-0.80	1.40	-3.57 – 1.97	-0.57	128.32	0.567
Age	-0.21	0.07	-0.36 – -0.07	-2.93	128.82	0.004

Probiotic [Treatment]	-3.35	1.29	-5.90 – -0.81	-2.60	216.08	0.010
Health	-2.76	0.60	-3.94 – -1.59	-4.62	216.83	<0.001
Time [Post]	-0.81	0.79	-2.37 – 0.74	-1.03	248.17	0.305
Time [fw]	-1.26	0.81	-2.86 – 0.34	-1.55	251.57	0.122
Probiotic [Treatment] ×× Health	1.94	0.91	0.15 – 3.74	2.14	216.42	0.034
Probiotic [Treatment] × Time [Post]	2.00	1.12	-0.20 – 4.20	1.79	248.57	0.075
Probiotic [Treatment] × Time [fw]	3.17	1.14	0.93 – 5.42	2.79	251.21	0.006
Health × Time [Post]	1.39	0.52	0.38 – 2.41	2.70	248.17	0.007
Health × Time [fw]	1.66	0.52	0.63 – 2.68	3.18	251.30	0.002
(Probiotic [Treatment] × Health) × Time [Post]	-2.15	0.79	-3.70 – -0.60	-2.73	248.22	0.007
(Probiotic [Treatment] × Health) × Time [fw]	-1.42	0.79	-2.98 – 0.13	-1.80	250.35	0.073
**Random Effects**						
σ^2	20.58					
$\tau_{00\ Subject}$	34.82					
ICC	0.63					
$N_{Subject}$	135					
Observations	391					
Marginal R^2 / Conditional R^2	0.154 / 0.686					

Table S12. Three-way interaction model for healthy behavior in SF36_MEN.

	SF36_MEN					
Predictors	*beta coeff.*	*Std. Error*	*CI*	*t statistic*	*df*	*p*
(Intercept)	-1.47	1.73	-4.89 – 1.94	-0.85	230.92	0.397
Sex [Male]	4.17	2.46	-0.70 – 9.04	1.69	129.09	0.093
Age	0.42	0.13	0.17 – 0.68	3.30	128.74	0.001
Probiotic [Treatment]	1.56	2.35	-3.07 – 6.19	0.66	241.84	0.508
Health	4.96	1.08	2.82 – 7.10	4.57	241.10	<0.001
Time [Post]	-0.60	1.61	-3.77 – 2.57	-0.37	253.60	0.710
Time [fw]	0.02	1.64	-3.20 – 3.24	0.01	255.20	0.992
Probiotic [Treatment] × Health	-0.20	1.65	-3.46 – 3.06	-0.12	241.89	0.906
Probiotic [Treatment] × Time [Post]	0.79	2.27	-3.67 – 5.26	0.35	253.59	0.727
Probiotic [Treatment] × Time [fw]	-0.58	2.30	-5.10 – 3.95	-0.25	254.92	0.801
Health × Time [Post]	-2.03	1.04	-4.07 – 0.02	-1.95	253.09	0.052
Health × Time [fw]	-2.57	1.05	-4.63 – -0.51	-2.46	253.86	0.015
(Probiotic [Treatment] × Health) × Time [Post]	1.84	1.59	-1.29 – 4.96	1.16	253.09	0.248
(Probiotic [Treatment] ×	1.01	1.59	-2.13 – 4.14	0.63	253.45	0.529

Health) × Time [fw]	

Random Effects

σ^2	85.82
$\tau_{00\ Subject}$	100.33
ICC	0.54
$N_{Subject}$	135
Observations	396
Marginal R^2 / Conditional R^2	0.213 / 0.637

Table S13. Three-way interaction model for healthy behavior in SF36_PHY.

	SF36_PHY					
Predictors	*beta coeff.*	*Std. Error*	*CI*	*t statistic*	*df*	*p*
(Intercept)	0.30	1.12	-1.91 – 2.52	0.27	216.58	0.787
Sex [Male]	2.04	1.63	-1.18 – 5.26	1.25	128.37	0.213
Age	0.03	0.08	-0.13 – 0.20	0.39	127.56	0.698
Probiotic [Treatment]	0.99	1.53	-2.04 – 4.01	0.64	230.51	0.521
Health	3.16	0.70	1.77 – 4.55	4.49	227.61	<0.001
Time [Post]	-0.54	0.99	-2.50 – 1.41	-0.55	245.56	0.583
Time [fw]	0.02	1.01	-1.96 – 2.01	0.02	246.95	0.982
Probiotic [Treatment] × Health	-0.33	1.08	-2.46 – 1.80	-0.30	231.70	0.762

Probiotic [Treatment] × Time [Post]	-0.42	1.41	-3.20 – 2.36	-0.30	246.32	0.768
Probiotic [Treatment] × Time [fw]	-2.47	1.42	-5.28 – 0.33	-1.74	247.65	0.084
Health × Time [Post]	-1.36	0.64	-2.63 – -0.10	-2.12	245.67	0.035
Health × Time [fw]	-1.14	0.66	-2.43 – 0.15	-1.74	247.47	0.084
(Probiotic [Treatment] × Health) × Time [Post]	1.69	1.00	-0.28 – 3.66	1.69	247.27	0.092
(Probiotic [Treatment] × Health) × Time [fw]	2.02	1.00	0.06 – 3.98	2.03	246.69	0.043
**Random Effects**						
σ^2	31.86					
$\tau_{00\ Subject}$	45.48					
ICC	0.59					
N $_{Subject}$	135					
Observations	387					
Marginal R^2 / Conditional R^2	0.191 / 0.667					

Table S14. Three-way interaction model for healthy behavior in MAIA.

	MAIA					
Predictors	*beta coeff.*	*Std. Error*	*CI*	*t statistic*	*df*	*p*
(Intercept)	-0.05	0.08	-0.22 – 0.11	-0.65	185.90	0.517
Sex [Male]	0.03	0.13	-0.22 – 0.28	0.23	128.99	0.820
Age	0.00	0.01	-0.01 – 0.02	0.76	128.85	0.448
Probiotic [Treatment]	0.06	0.11	-0.16 – 0.28	0.54	193.68	0.591
Health	0.11	0.05	0.01 – 0.21	2.13	192.02	0.034
Time [Post]	-0.01	0.06	-0.13 – 0.11	-0.18	252.34	0.861
Time [fw]	-0.06	0.06	-0.19 – 0.06	-1.04	253.30	0.298
Probiotic [Treatment] × Health	0.27	0.08	0.11 – 0.42	3.36	200.33	0.001
Probiotic [Treatment] × Time [Post]	0.07	0.09	-0.10 – 0.24	0.82	252.65	0.414
Probiotic [Treatment] × Time [fw]	0.19	0.09	0.02 – 0.37	2.21	253.35	0.028
Health × Time [Post]	-0.06	0.04	-0.13 – 0.02	-1.46	252.04	0.146
Health × Time [fw]	-0.01	0.04	-0.09 – 0.06	-0.35	252.50	0.726
(Probiotic [Treatment] × Health) × Time [Post]	-0.08	0.06	-0.20 – 0.05	-1.23	254.65	0.219
(Probiotic [Treatment] × Health) × Time [fw]	-0.09	0.06	-0.21 – 0.03	-1.41	253.50	0.160

Random Effects

σ^2	0.12
$\tau_{00\ Subject}$	0.29
ICC	0.70
$N_{Subject}$	135
Observations	395
Marginal R^2 / Conditional R^2	0.191 / 0.761

Table S15. Three-way interaction model for healthy behavior in FFMQ.

	FFMQ					
Predictors	*beta coeff.*	*Std. Error*	*CI*	*t statistic*	*df*	*p*
(Intercept)	-1.04	2.29	-5.55 – 3.47	-0.45	185.04	0.650
Sex [Male]	2.14	3.48	-4.74 – 9.02	0.62	128.98	0.539
Age	0.38	0.18	0.02 – 0.74	2.11	128.77	0.037
Probiotic [Treatment]	1.44	3.08	-4.63 – 7.51	0.47	190.48	0.640
Health	4.67	1.43	1.84 – 7.49	3.25	196.01	0.001
Time [Post]	-0.23	1.66	-3.50 – 3.04	-0.14	252.63	0.889
Time [fw]	-0.22	1.69	-3.54 – 3.11	-0.13	253.55	0.899
Probiotic [Treatment] × Health	1.26	2.17	-3.03 – 5.55	0.58	192.55	0.563
Probiotic [Treatment] × Time [Post]	0.16	2.33	-4.44 – 4.75	0.07	252.48	0.946

Probiotic [Treatment] × Time [fw]	0.75	2.37	-3.91 – 5.41	0.32	253.24	0.752
Health × Time [Post]	-1.98	1.09	-4.12 – 0.16	-1.83	253.13	0.069
Health × Time [fw]	-1.36	1.10	-3.51 – 0.80	-1.24	253.58	0.217
(Probiotic [Treatment] × Health) × Time [Post]	3.93	1.64	0.70 – 7.17	2.39	252.51	0.017
(Probiotic [Treatment] × Health) × Time [fw]	3.49	1.65	0.24 – 6.74	2.11	252.73	0.035
**Random Effects**						
σ^2	90.30					
$\tau_{00\ \text{Subject}}$	227.46					
ICC	0.72					
N_{Subject}	135					
Observations	395					
Marginal R^2 / Conditional R^2	0.198 / 0.772					

Table S16. Three-way interaction model for risky behavior in RYFF.

	RYFF					
Predictors	*beta coeff.*	*Std. Error*	*CI*	*t statistic*	*df*	*p*
(Intercept)	-0.05	0.07	-0.19 – 0.09	-0.70	183.42	0.483
Sex [Male]	0.05	0.11	-0.16 – 0.26	0.45	129.06	0.656
Age	0.02	0.01	0.01 – 0.03	3.15	128.87	0.002

Probiotic [Treatment]	0.11	0.09	-0.08 – 0.29	1.16	190.69	0.247
Risk	-0.13	0.05	-0.24 – -0.03	-2.47	189.69	0.015
Time [Post]	-0.02	0.05	-0.12 – 0.08	-0.43	253.03	0.666
Time [fw]	-0.06	0.05	-0.16 – 0.04	-1.14	254.18	0.257
Probiotic [Treatment] × Risk	0.07	0.07	-0.07 – 0.20	0.95	190.35	0.344
Probiotic [Treatment] × Time [Post]	0.01	0.07	-0.13 – 0.15	0.18	253.29	0.859
Probiotic [Treatment] × Time [fw]	0.09	0.07	-0.05 – 0.23	1.28	254.21	0.203
Risk × Time [Post]	0.01	0.04	-0.07 – 0.09	0.16	253.03	0.874
Risk × Time [fw]	-0.02	0.04	-0.10 – 0.07	-0.39	254.47	0.700
(Probiotic [Treatment] × Risk) × Time [Post]	0.02	0.05	-0.08 – 0.13	0.42	254.31	0.672
(Probiotic [Treatment] × Risk) × Time [fw]	0.00	0.05	-0.10 – 0.11	0.02	254.69	0.984
**Random Effects**						
σ^2	0.08					
$\tau_{00\ Subject}$	0.21					
ICC	0.72					
$N_{Subject}$	135					
Observations	396					
Marginal R^2 / Conditional R^2	0.133 / 0.754					

Table S17. Three-way interaction model for risky behavior in SWLS.

	SWLS					
Predictors	*beta coeff.*	*Std. Error*	*CI*	*t statistic*	*df*	*p*
(Intercept)	-0.20	0.44	-1.07 – 0.66	-0.46	197.01	0.643
Sex [Male]	-0.41	0.66	-1.71 – 0.89	-0.62	128.88	0.535
Age	0.09	0.03	0.02 – 0.16	2.63	128.27	0.010
Probiotic [Treatment]	0.97	0.60	-0.21 – 2.15	1.62	207.02	0.106
Risk	-0.53	0.35	-1.22 – 0.15	-1.53	204.75	0.127
Time [Post]	-0.22	0.35	-0.91 – 0.47	-0.62	249.14	0.535
Time [fw]	-0.08	0.36	-0.79 – 0.62	-0.24	250.55	0.814
Probiotic [Treatment] × Risk	0.42	0.44	-0.45 – 1.29	0.95	206.58	0.344
Probiotic [Treatment] × Time [Post]	0.40	0.50	-0.59 – 1.38	0.79	249.32	0.428
Probiotic [Treatment] × Time [fw]	0.08	0.51	-0.92 – 1.07	0.15	251.67	0.882
Risk × Time [Post]	0.03	0.29	-0.53 – 0.60	0.12	249.14	0.906
Risk × Time [fw]	-0.13	0.30	-0.71 – 0.45	-0.44	250.91	0.658
(Probiotic [Treatment] × Risk) × Time [Post]	0.02	0.37	-0.70 – 0.74	0.05	249.15	0.960
(Probiotic [Treatment] ×	0.08	0.38	-0.68 – 0.83	0.20	253.17	0.843

Risk) × Time [fw]	

Random Effects

σ^2	4.09
$\tau_{00\ Subject}$	7.82
ICC	0.66
$N_{Subject}$	135
Observations	392
Marginal R^2 / Conditional R^2	0.082 / 0.685

Table S18. Three-way interaction model for risky behavior in STAI.

	STAI					
Predictors	*beta coeff.*	*Std. Error*	*CI*	*t statistic*	*df*	*p*
(Intercept)	1.24	0.96	-0.66 – 3.14	1.29	262.20	0.200
Sex [Male]	-1.67	1.31	-4.26 – 0.92	-1.28	129.24	0.203
Age	-0.14	0.07	-0.27 – -0.00	-2.02	128.82	0.045
Probiotic [Treatment]	-1.77	1.31	-4.36 – 0.82	-1.35	276.26	0.179
Risk	1.50	0.77	-0.01 – 3.01	1.96	275.15	0.051
Time [Post]	-0.38	0.98	-2.31 – 1.56	-0.38	254.12	0.703
Time [fw]	0.26	1.00	-1.71 – 2.24	0.26	256.65	0.795
Probiotic [Treatment] × Risk	-0.02	0.99	-1.97 – 1.93	-0.02	286.05	0.983

Probiotic [Treatment] × Time [Post]	0.74	1.39	-1.99 – 3.48	0.53	254.39	0.593
Probiotic [Treatment] × Time [fw]	-0.56	1.41	-3.34 – 2.22	-0.39	256.63	0.693
Risk × Time [Post]	0.49	0.81	-1.11 – 2.08	0.60	254.12	0.547
Risk × Time [fw]	0.27	0.83	-1.36 – 1.90	0.33	257.29	0.745
(Probiotic [Treatment] × Risk) × Time [Post]	-1.04	1.04	-3.09 – 1.01	-1.00	256.62	0.320
(Probiotic [Treatment] × Risk) × Time [fw]	0.19	1.07	-1.92 – 2.30	0.18	260.42	0.858
**Random Effects**						
σ^2	32.30					
$\tau_{00\ Subject}$	25.52					
ICC	0.44					
$N_{Subject}$	135					
Observations	397					
Marginal R^2 / Conditional R^2	0.116 / 0.506					

Table S19. Three-way interaction model for risky behavior in DERS.

	DERS					
Predictors	*beta coeff.*	*Std. Error*	*CI*	*t statistic*	*df*	*p*
(Intercept)	0.32	1.29	-2.22 – 2.87	0.25	208.93	0.802
Sexo [Male]	-2.09	1.89	-5.82 – 1.64	-1.11	128.16	0.269
Edad	-0.36	0.10	-0.55 – -0.16	-3.60	128.30	<0.001
Probiotico [Treatment]	-1.28	1.75	-4.73 – 2.17	-0.73	219.96	0.466
Risk	0.51	1.02	-1.50 – 2.51	0.50	215.72	0.618
Time [Post]	0.48	1.09	-1.67 – 2.62	0.44	248.04	0.662
Time [fw]	0.10	1.11	-2.09 – 2.29	0.09	249.69	0.931
Probiotico [Treatment] × Risk	1.78	1.31	-0.81 – 4.37	1.35	227.98	0.177
Probiotico [Treatment] × Time [Post]	0.13	1.54	-2.91 – 3.17	0.09	247.81	0.932
Probiotico [Treatment] × Time [fw]	0.57	1.57	-2.52 – 3.66	0.36	250.34	0.717
Risk × Time [Post]	0.84	0.89	-0.92 – 2.59	0.94	247.52	0.349
Risk × Time [fw]	1.28	0.93	-0.55 – 3.10	1.38	250.37	0.169
(Probiotico [Treatment] × Risk) × Time [Post]	-1.25	1.17	-3.55 – 1.04	-1.07	247.73	0.283
(Probiotico [Treatment] × Risk) × Time [fw]	-1.38	1.20	-3.74 – 0.99	-1.15	255.86	0.253

Random Effects

σ^2	38.84
$\tau_{00\ Subject}$	62.77
ICC	0.62
$N_{Subject}$	135
Observations	390
Marginal R^2 / Conditional R^2	0.136 / 0.670

Table S20. Three-way interaction model for risky behavior in PANAS_POS.

	PANAS_POS					
Predictors	*beta coeff.*	*Std. Error*	*CI*	*t statistic*	*df*	*p*
(Intercept)	-0.43	0.88	-2.16 – 1.30	-0.49	257.76	0.625
Sex [Male]	1.58	1.19	-0.77 – 3.94	1.33	128.48	0.186
Age	0.09	0.06	-0.04 – 0.21	1.40	128.48	0.165
Probiotic [Treatment]	0.73	1.19	-1.62 – 3.07	0.61	269.48	0.543
Risk	0.38	0.72	-1.05 – 1.80	0.52	285.00	0.602
Time [Post]	-0.68	0.89	-2.42 – 1.07	-0.76	252.62	0.447
Time [fw]	-0.58	0.90	-2.35 – 1.18	-0.65	255.83	0.516
Probiotic [Treatment] × Risk	-1.54	0.92	-3.34 – 0.27	-1.68	288.30	0.095
Probiotic [Treatment] ×	1.30	1.24	-1.14 – 3.75	1.05	252.24	0.295

Time [Post]						
Probiotic [Treatment] × Time [fw]	0.57	1.26	-1.90 – 3.05	0.45	254.78	0.650
Risk × Time [Post]	-1.72	0.76	-3.22 – -0.23	-2.27	251.41	0.024
Risk × Time [fw]	-1.74	0.77	-3.25 – -0.23	-2.26	265.11	0.024
(Probiotic [Treatment] × Risk) × Time [Post]	2.28	0.96	0.40 – 4.17	2.39	253.60	0.018
(Probiotic [Treatment] × Risk) × Time [fw]	1.62	0.97	-0.30 – 3.54	1.66	263.89	0.097
**Random Effects**						
σ^2	25.39					
$\tau_{00\ Subject}$	21.72					
ICC	0.46					
$N_{Subject}$	135					
Observations	394					
Marginal R^2 / Conditional R^2	0.072 / 0.500					

Table S21. Three-way interaction model for risky behavior in PANAS_NEG.

	PANAS_NEG					
Predictors	*beta coeff.*	*Std. Error*	*CI*	*t statistic*	*df*	*p*
(Intercept)	1.59	0.95	-0.27 – 3.46	1.68	209.42	0.094
Sex [Male]	-1.25	1.38	-3.99 – 1.48	-0.91	128.11	0.365
Age	-0.22	0.07	-0.36 – -0.08	-3.02	128.63	0.003
Probiotic [Treatment]	-3.62	1.28	-6.15 – -1.08	-2.81	220.54	0.005
Risk	3.48	0.75	2.00 – 4.95	4.65	218.47	<0.001
Time [Post]	-0.90	0.80	-2.48 – 0.67	-1.13	247.19	0.258
Time [fw]	-1.36	0.82	-2.97 – 0.25	-1.66	250.67	0.098
Probiotic [Treatment] × Risk	-2.47	0.96	-4.36 – -0.58	-2.58	226.92	0.011
Probiotic [Treatment] × Time [Post]	2.00	1.13	-0.23 – 4.23	1.77	247.79	0.079
Probiotic [Treatment] × Time [fw]	3.23	1.15	0.95 – 5.50	2.80	250.52	0.006
Risk × Time [Post]	-0.66	0.66	-1.95 – 0.64	-1.00	247.19	0.320
Risk × Time [fw]	-1.86	0.68	-3.19 – -0.52	-2.74	251.30	**0.007**
(Probiotic [Treatment] × Risk) × Time [Post]	0.06	0.85	-1.60 – 1.73	0.07	248.89	0.942
(Probiotic [Treatment] × Risk) × Time [fw]	1.05	0.87	-0.66 – 2.77	1.21	252.45	0.228

Random Effects

σ^2	20.98
$\tau_{00\ Subject}$	33.61
ICC	0.62
$N_{Subject}$	135
Observations	390
Marginal R^2 / Conditional R^2	0.166 / 0.680

Table S22. Three-way interaction model for risky behavior in SF36_MEN.

	SF36_MEN					
Predictors	*beta coeff.*	*Std. Error*	*CI*	*t statistic*	*df*	*p*
(Intercept)	-2.03	1.81	-5.60 – 1.54	-1.12	220.12	0.264
Sex [Male]	5.21	2.61	0.05 – 10.38	2.00	128.93	0.048
Age	0.44	0.14	0.17 – 0.71	3.23	128.69	0.002
Probiotic [Treatment]	1.91	2.46	-2.94 – 6.75	0.78	231.12	0.439
Risk	-4.72	1.43	-7.55 – -1.90	-3.29	230.10	0.001
Time [Post]	-0.47	1.61	-3.65 – 2.70	-0.29	252.54	0.770
Time [fw]	0.17	1.64	-3.05 – 3.40	0.10	253.89	0.917
Probiotic [Treatment] × Risk	1.71	1.84	-1.92 – 5.34	0.93	240.21	0.355
Probiotic [Treatment] × Time [Post]	0.91	2.28	-3.58 – 5.39	0.40	252.73	0.691

Probiotic [Treatment] × Time [fw]	-0.49	2.31	-5.04 – 4.05	-0.21	253.88	0.830
Risk × Time [Post]	1.67	1.32	-0.93 – 4.27	1.26	252.07	0.207
Risk × Time [fw]	1.48	1.36	-1.19 – 4.15	1.09	254.35	0.277
(Probiotic [Treatment] × Risk) × Time [Post]	0.68	1.70	-2.67 – 4.04	0.40	253.88	0.688
(Probiotic [Treatment] × Risk) × Time [fw]	0.44	1.75	-3.01 – 3.88	0.25	256.63	0.804
**Random Effects**						
σ^2	86.03					
$\tau_{00\ Subject}$	116.42					
ICC	0.58					
$N_{Subject}$	135					
Observations	395					
Marginal R^2 / Conditional R^2	0.142 / 0.635					

Table S23. Three-way interaction model for risky behavior in SF36_PHY.

	SF36_PHY					
Predictors	*beta coeff.*	*Std. Error*	*CI*	*t statistic*	*df*	*p*
(Intercept)	-0.00	1.20	-2.37 – 2.36	-0.00	205.89	0.998
Sex [Male]	2.70	1.76	-0.78 – 6.19	1.53	128.39	0.128
Age	0.03	0.09	-0.15 – 0.22	0.37	127.71	0.715
Probiotic [Treatment]	1.35	1.64	-1.87 – 4.57	0.83	218.98	0.410
Risk	-2.69	0.95	-4.57 – -0.82	-2.84	215.50	0.005
Time [Post]	-0.52	1.00	-2.49 – 1.46	-0.51	244.46	0.609
Time [fw]	0.07	1.02	-1.94 – 2.08	0.07	245.49	0.945
Probiotic [Treatment] × Risk	1.68	1.22	-0.74 – 4.09	1.37	227.59	0.172
Probiotic [Treatment] × Time [Post]	-0.36	1.43	-3.18 – 2.46	-0.25	245.01	0.804
Probiotic [Treatment] × Time [fw]	-2.44	1.45	-5.28 – 0.41	-1.69	246.32	0.093
Risk × Time [Post]	0.84	0.82	-0.78 – 2.47	1.03	244.08	0.306
Risk × Time [fw]	0.18	0.84	-1.48 – 1.84	0.22	245.70	0.829
(Probiotic [Treatment] × Risk) × Time [Post]	-1.23	1.07	-3.33 – 0.88	-1.15	245.96	0.252
(Probiotic [Treatment] × Risk) × Time [fw]	-0.78	1.09	-2.93 – 1.38	-0.71	248.22	0.479

Random Effects

σ^2	32.59
$\tau_{00\ Subject}$	55.09
ICC	0.63
$N_{Subject}$	135
Observations	386
Marginal R^2 / Conditional R^2	0.087 / 0.661

Table S24. Three-way interaction model for risky behavior in MAIA.

	MAIA					
Predictors	*beta coeff.*	*Std. Error*	*CI*	*t statistic*	*df*	*p*
(Intercept)	-0.07	0.09	-0.23 – 0.10	-0.77	185.49	0.444
Sex [Male]	0.05	0.13	-0.21 – 0.31	0.38	129.41	0.701
Age	0.00	0.01	-0.01 – 0.02	0.46	128.55	0.647
Probiotic [Treatment]	0.12	0.12	-0.11 – 0.35	1.05	192.75	0.294
Risk	-0.12	0.07	-0.25 – 0.02	-1.72	191.95	0.086
Time [Post]	-0.01	0.06	-0.13 – 0.12	-0.13	251.40	0.900
Time [fw]	-0.06	0.06	-0.19 – 0.06	-0.99	252.30	0.325
Probiotic [Treatment] × Risk	-0.07	0.09	-0.24 – 0.10	-0.82	199.94	0.414
Probiotic [Treatment] × Time [Post]	0.00	0.09	-0.18 – 0.18	0.01	251.71	0.992

Probiotic [Treatment] × Time [fw]	0.15	0.09	-0.02 – 0.33	1.70	252.88	0.091
Risk × Time [Post]	0.03	0.05	-0.07 – 0.13	0.58	251.10	0.561
Risk × Time [fw]	0.03	0.05	-0.07 – 0.14	0.59	252.60	0.553
(Probiotic [Treatment] × Risk) × Time [Post]	-0.07	0.07	-0.20 – 0.07	-0.97	253.07	0.335
(Probiotic [Treatment] × Risk) × Time [fw]	-0.03	0.07	-0.17 – 0.10	-0.47	258.25	0.639
**Random Effects**						
σ^2	0.13					
$\tau_{00\ Subject}$	0.32					
ICC	0.70					
$N_{Subject}$	135					
Observations	394					
Marginal R^2 / Conditional R^2	0.118 / 0.740					

Table S25. Three-way interaction model for risky behavior in FFMQ.

	FFMQ					
Predictors	*beta coeff.*	*Std. Error*	*CI*	*t statistic*	*df*	*p*
(Intercept)	-1.53	2.37	-6.20 – 3.14	-0.65	179.91	0.519
Sex [Male]	3.59	3.62	-3.58 – 10.75	0.99	129.03	0.324
Age	0.35	0.19	-0.03 – 0.72	1.82	128.87	0.071
Probiotic [Treatment]	2.26	3.19	-4.03 – 8.54	0.71	185.79	0.480
Risk	-4.39	1.86	-8.06 – -0.71	-2.35	185.80	0.020
Time [Post]	-0.31	1.67	-3.59 – 2.98	-0.18	253.30	0.854
Time [fw]	-0.26	1.69	-3.60 – 3.07	-0.16	254.11	0.876
Probiotic [Treatment] × Risk	-0.35	2.35	-4.99 – 4.29	-0.15	185.78	0.882
Probiotic [Treatment] × Time [Post]	0.29	2.35	-4.33 – 4.92	0.12	253.30	0.901
Probiotic [Treatment] × Time [fw]	0.89	2.38	-3.79 – 5.58	0.38	253.98	0.707
Risk × Time [Post]	0.11	1.37	-2.58 – 2.80	0.08	253.02	0.938
Risk × Time [fw]	-0.99	1.40	-3.75 – 1.77	-0.71	254.39	0.480
(Probiotic [Treatment] × Risk) × Time [Post]	0.15	1.72	-3.24 – 3.55	0.09	253.03	0.929
(Probiotic [Treatment] × Risk) × Time [fw]	1.14	1.77	-2.36 – 4.63	0.64	254.46	0.522

Random Effects

σ^2	91.90
$\tau_{00\ Subject}$	249.59
ICC	0.73
$N_{Subject}$	135
Observations	396
Marginal R^2 / Conditional R^2	0.141 / 0.769
