# Supplementary figures and images for: Psychobiotic Effects on Anxiety Are Modulated by Lifestyle Behaviors: A Randomized Placebo-Controlled Trial on Healthy Adults
Source: Nutrients. 2023 Mar 31;15(7):1706. doi: 10.3390/nu15071706 (PMC10096963; doi:10.3390/nu15071706)

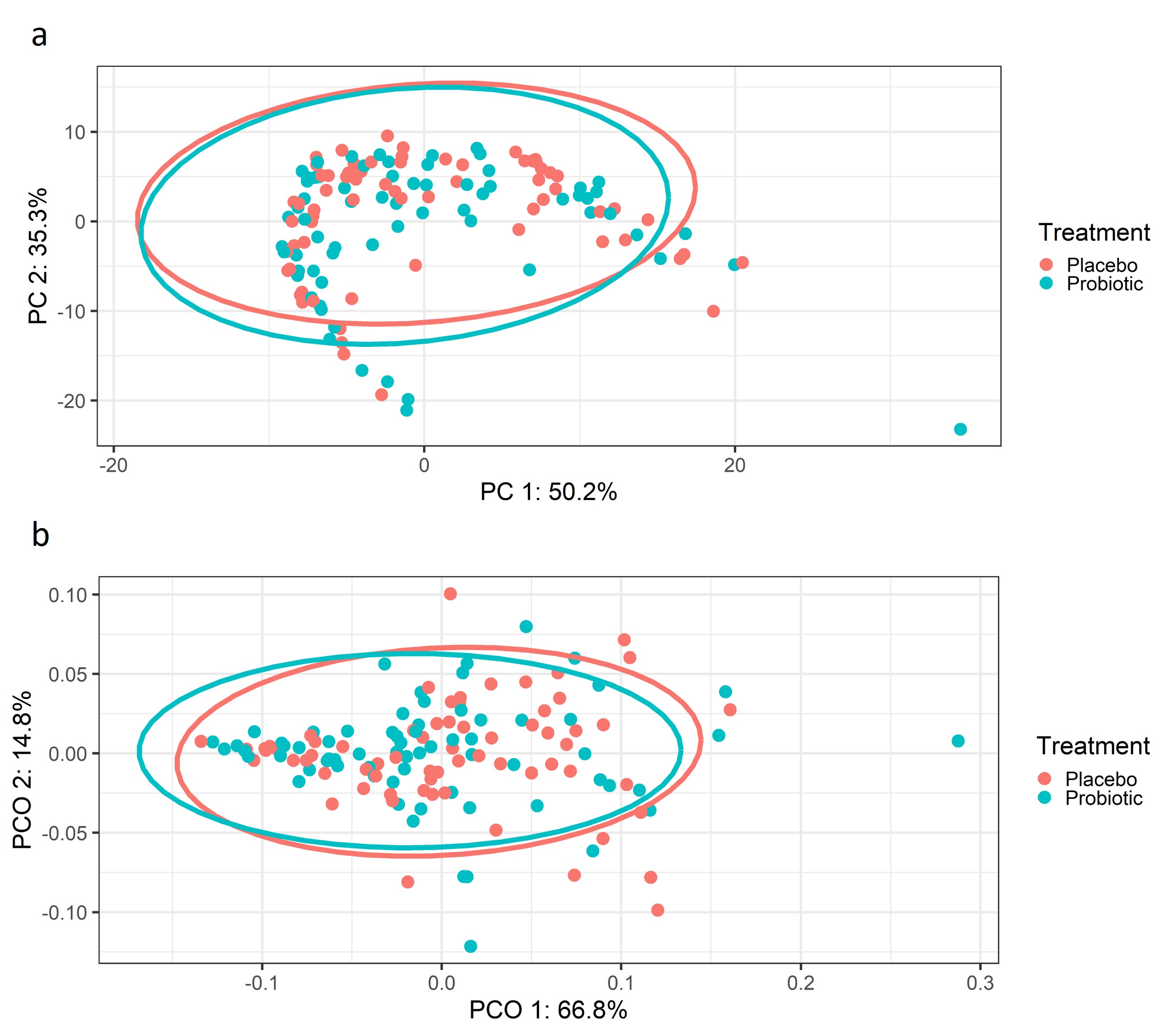

Supplement: Supplementary file 1 [file nutrients-15-01706-s001.zip › Figure S1.jpg]

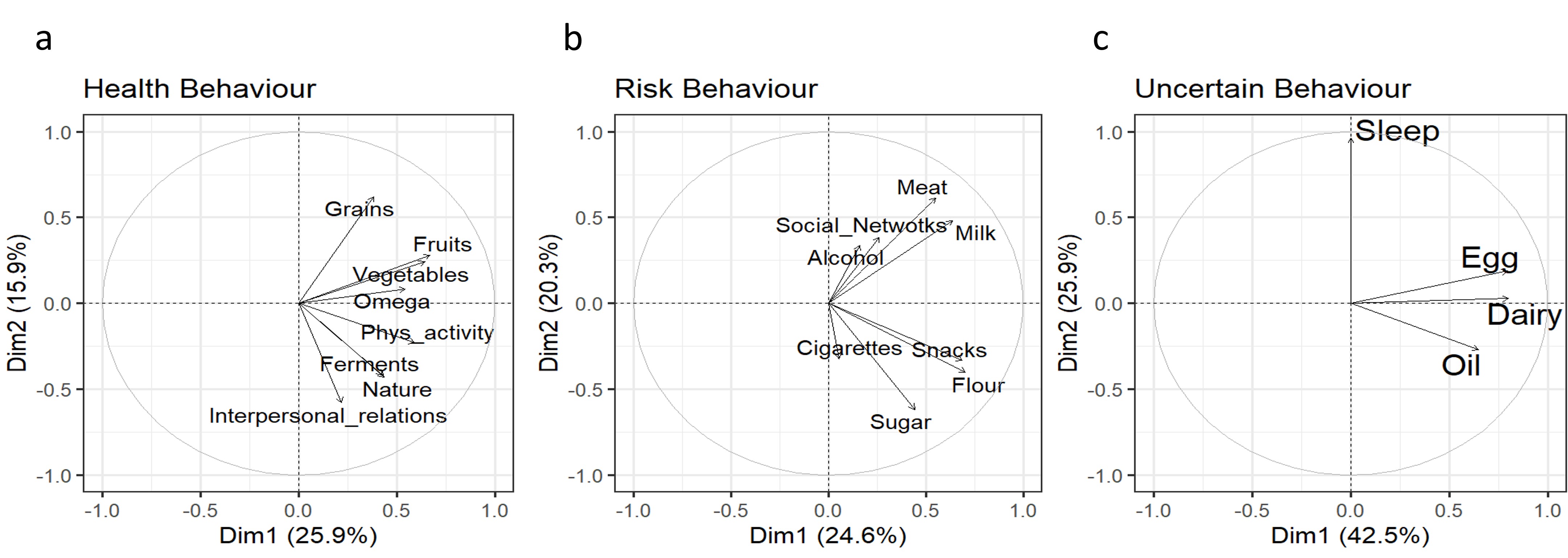

Supplement: Supplementary file 1 [file nutrients-15-01706-s001.zip › Figure S2.jpg]

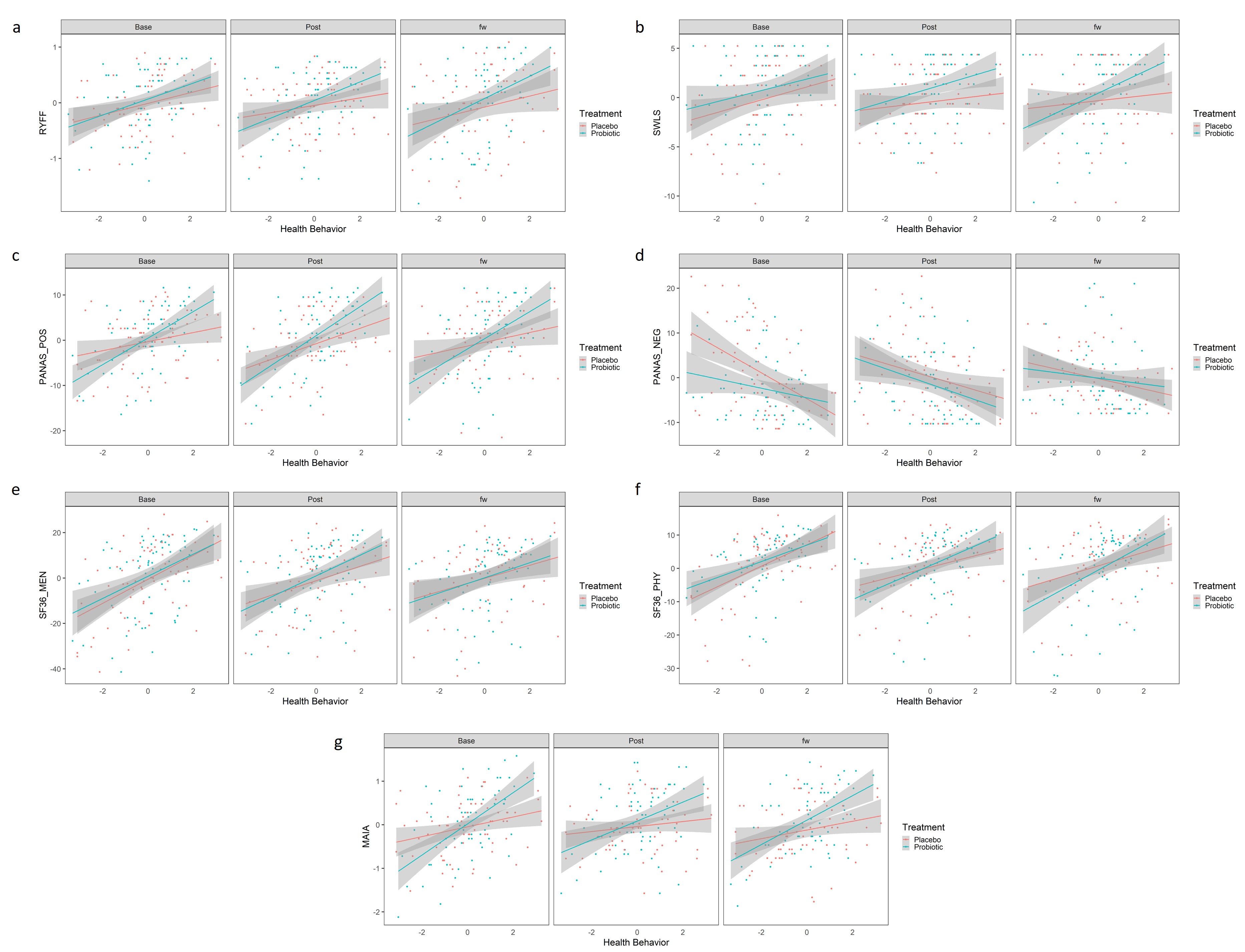

Supplement: Supplementary file 1 [file nutrients-15-01706-s001.zip › Figure S3.jpg]
